# Acute Kidney Injury Secondary to Pegylated Liposomal Doxorubicin-Associated Renal-limited Thrombotic Microangiopathy

**DOI:** 10.1016/j.xkme.2025.100998

**Published:** 2025-03-19

**Authors:** Tarek S. Karam, Mrinalini Sarkar, Jonathan E. Zuckerman

**Affiliations:** 1 Department of Nephrology, University of California, Los Angeles Medical Center, Los Angeles, CA; 2 Department of Pathology and Laboratory Medicine, University of California, Los Angeles Medical Center, Los Angeles, CA

**Keywords:** Doxil, kidney biopsy, onconephrology, pegylated liposomal doxorubicin, thrombotic microangiopathy (TMA), renal pathology

## Abstract

The emergence of pegylated liposomal doxorubicin (PLD) as a preferred treatment for various malignancies, because of its reduced cardiotoxicity compared with conventional doxorubicin, has raised significant interest. However, the association between PLD and thrombotic microangiopathy (TMA) remains a concerning and relatively rare complication. Here, we present the case of an 80-year-old man with metastatic Kaposi sarcoma who underwent extended PLD monotherapy, subsequently developing kidney-limited TMA demonstrated on kidney biopsy. This led to acute kidney injury necessitating hemodialysis. The patient’s clinical history, laboratory, and kidney biopsy data supported PLD chemotherapy as the primary etiologic factor for the observed kidney-limited TMA, an insidious condition with poor prognosis. This report highlights the need for vigilance and early kidney biopsy in patients with rising serum creatinine concentrations or worsening proteinuria/hematuria during PLD therapy. Understanding the mechanisms underlying PLD-induced TMA, likely involving reactive oxygen species-mediated endothelial dysfunction and platelet aggregation, remains a crucial area for future research to optimize monitoring and management strategies for this rare yet severe complication associated with PLD therapy.

## Introduction

Doxorubicin, a potent anthracycline chemotherapy, has been a cornerstone in treating diverse malignancies. It intercalates with DNA and impedes topoisomerase activity, disrupting replication and causing cytotoxicity in rapidly dividing cells. Because of significant side effects such as myelosuppression, acute interstitial nephritis, and cardiotoxicity leading to heart failure, there has been a shift toward use of pegylated liposomal doxorubicin (PLD).^1^

Formulation of doxorubicin within a pegylated liposome results in an extended serum half-life as well as enhanced therapeutic efficacy by improving tumor targeting. Concurrently, the incidence and severity of certain adverse effects typically associated with conventional doxorubicin usage, particularly cardiotoxicity and bone marrow suppression, are reduced with PLD treatment. PLD is widely applied in various cancer treatments because of its altered pharmacokinetic and pharmacodynamic properties, making it an important agent in the oncologic therapeutic arsenal.^2^

Thrombotic microangiopathy (TMA) encompasses a spectrum of disorders resulting from endothelial cell damage that can result in hemolytic anemia, thrombocytopenia, and end organ damage, including kidney injury.^3^ Only rare reports of PLD-associated TMA have been documented, often in the setting of concurrent treatment with drugs more commonly associated with TMA, such as gemcitabine.^4^ Our report highlights a case of kidney-limited TMA in a patients with Kaposi sarcoma undergoing PLD monotherapy.

## Case Report

**Figure 1:**
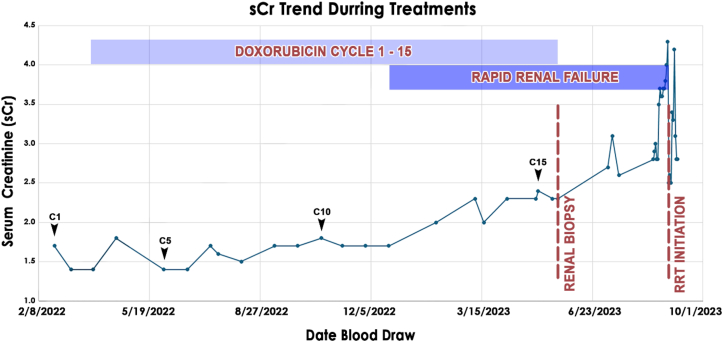
Progression of sCr relative to PLD exposure. Slow progression of sCr over 15 cycles of 20 mg/m^2^ PLD. PLD was discontinued after kidney biopsy findings were obtained, with ongoing worsening of kidney function to ESRD requiring hemodialysis (RRT). sCr fluctuations after RRT initiation was a function of RRT and sCr removal. Some kidney function was recovered with good urine output, and initially RRT was discontinued. However, because of low creatinine clearance, the patient was reinitiated twice weekly for clearance (not shown). Abbreviations: ESRD, end-stage kidney disease, PLD, pegylated liposomal doxorubicin; RRT, renal replacement therapy; sCr, serum creatinine.

An 80-year-old man with controlled type 2 diabetes, hypertension, hyperlipidemia, chronic kidney disease stage 3A, and a 12-month history of metastatic human herpesvirus 8-positive Kaposi sarcoma presented to University of California, Los Angeles Health Nephrology Clinic for evaluation of elevated serum creatinine (sCr) concentrations and hypertension. He was receiving 20 mg/m^2^ PLD every 3 weeks for Kaposi sarcoma involving the skin and lymph nodes. Baseline sCr was 1.4 mg/dL with an estimated glomerular filtration rate of 55 mL/min/1.73 m^2^ and minimal proteinuria. Treatment initially reduced lymphadenopathy with stable complete blood count and comprehensive metabolic panel. However, at cycle 8, sCr increased to 1.7-1.8 mg/dL, worsening to 2.3-2.4 mg/dL by cycle 15 (Fig 1; PLD cumulative dose, 300 mg/m^2^). Nephrotic range proteinuria emerged after cycle 14, with an albumin-creatinine ratio of 3,779 mg/g, worsening to 5,308 mg/g by cycle 15 (Table 1).

**Table 1:** Clinical Laboratory Test Results

Laboratory Tests	Before Doxi	Doxi C1	Doxi C5	Doxi C10	Doxi C15	RRT Initiation	Ref Range
	10/18/2021	2/22/2022	6/1/2022	10/21/2022	5/5/2023	8/30/2023	
Hemoglobin, g/dL	9.2	8.8	6.7	8.7	8.7	7.3	13.5-17.1
WBC count, ×10^9^ /L	6	4.4	9.9	3.1	5.7	6.65	4.16-9.95
Platelet count, ×10^9^ /L	172	314	193	192	215	168	143-398
Sodium, mmol/L	139	140	143	141	144	141	135-146
Potassium, mmol/L	4.2	3.64	4.1	4.2	3.8	3.7	3.6-5.3
Bicarbonate, mmol/L	26	26	27	23	24	24	20-30
sCr, mg/dL	1.4	1.55	1.4	1.8	2.3	4.35	0.6-1.3
SUN, mg/dL	24	22	27	30	33	84	22*-*Jul
Calcium, mg/dL	10.1	9.8	9.6	9	8.7	9.3	8.6-10.4
Phosphate, mg/dL					4.3	5.6	2.3-4.4
Albumin, g/dL	3.6	3.5	3.6	3.4	3.2	4.4	3.9-5.0
AST, U/L	11	16	10	11	19	14	13-62
ALT, U/L	10	9	7	7	9	12	08-70
ALP, U/L	53	51	57	50	73	46	37-113
Total bilirubin, mg/dL	0.6	0.5	0.7	0.4	0.4	0.8	01-1.2
INR	—	—	—	—	1.2	1	
PTT, s	—	—	—	—	35	34.8	24.4-36.2
Urinalysis							
Hematuria	0	0	0	0	1+	3+	<2
Protonuria	Trace	Negative	1+	—	3+	3+	Negative
Urinary sediment	—	—	—	—	—	—	—
Albumin-to-creatinine, mg/g	Negative	—	454.7	—	5,308	5,860	<30

*Note*: Conversion factors for units: serum creatinine concentrations in mg/dL to μmol/L, ×88; urea nitrogen in mg/dL to mmol/L, ×0.357.

Abbreviations: ALP, alkaline phosphatase; ALT, alanine aminotransferase; AST, aspartate aminotransferase; SUN, serum urea nitrogen; C, cycle; Doxi, pegylated liposomal doxorubicin; INR, international normalized ratio; PTT, partial thromboplastin time; RRT, renal replacement therapy; sCr, serum creatinine; WBC, white blood cell.

To investigate the cause of the acute kidney injury (AKI) and proteinuria, an extensive history and workup were conducted, followed by kidney biopsy. Peripheral smear showed no schistocytes or fragmented cells. Haptoglobin, platelets, lactate dehydrogenase, and bilirubin were within normal limits. Human immunodeficiency virus, hepatitis B/C, rapid plasma reagin, antinuclear antibody, phospholipase A2 receptor antibodies, and thrombospondin domain-containing 7A serologic studies were negative. Complement C3 and C4 levels were normal. Autoimmune-mediated and complement-mediated glomerulopathies, hemolytic uremic syndrome, and infections became lower on the differential. Membranous nephropathy, drug toxicity, or a paraneoplastic process could not be ruled out. Therefore, the patient underwent a kidney biopsy to elucidate the cause of the AKI and proteinuria.

**Figure 2:**
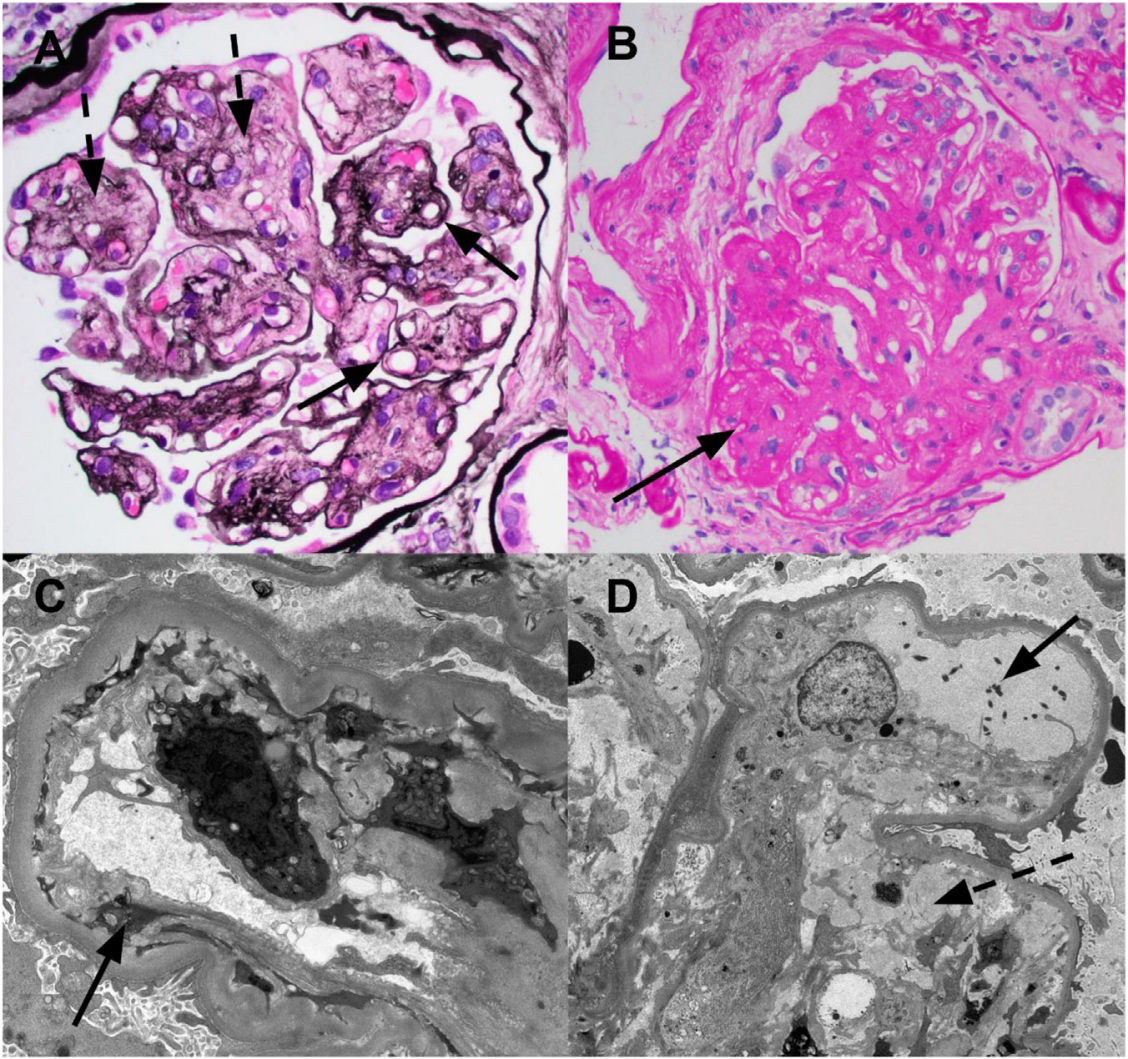
(A) Chronic active thrombotic microangiopathy pattern of injury with mesangiolysis (dashed arrow) and capillary wall double contour formation (solid arrow) (Jones silver stain; original magnification ×4000). (B) Lesion of segmental glomerulosclerosis (arrow) (periodic acid–Schiff stain; original magnification ×400). (C) Chronic endothelial cell injury with subendothelial widening, basement membrane duplication, and cellular interposition. Arrow points to area of subendothelial widening and cellular interposition (electron microscopy; original magnification, ×4800). (D) Active endothelial cell injury with mesangiolysis (dashed arrow) and few luminal fibrin tactoid (solid arrow) (electron microscopy; original magnification, ×1900).

By light microscopy, the glomeruli exhibited frequent mesangiolysis and double contour formation, as well as endothelial cell swelling. Mesangial regions exhibited early focal sclerosis with some degree of early nodule formation. There were no necrotizing lesions, crescents, or capillary wall spike/pinhole formations. At least one glomerulus was involved by segmental sclerosis. There was patchy acute tubular injury, and tubules exhibited thickened basement membranes. There was 40%-50% interstitial fibrosis/tubular atrophy. Immunofluorescence studies were negative for any immune complex deposition. Electron microscopy showed significant, frequent glomerular capillary wall double contour formation with cellular interposition and overlying endothelial cell swelling. Rare luminal fibrin tactoids were present. Additionally, there was segmental podocyte foot process effacement (40%) and thickened glomerular basement membranes (up to 780 nm). There were no tubuloreticular inclusions or electron-dense deposits (Fig 2). The absence of systemic TMA features, combined with the temporal relationship between PLD administration and kidney injury and an otherwise negative workup for an alternative etiology, supports PLD as the likely cause. The lack of thrombi on biopsy does not exclude a TMA diagnosis, because renal-limited TMA is a well-recognized entity characterized by endothelial cell injury without systemic microangiopathy. Finally, given the mesangial sclerosis, a component of diabetic glomerulopathy could not be reliably excluded, because mesangial sclerosis can also be a sequela of chronic microangiopathy.

A diagnosis of chronic active microangiopathic glomerular injury with secondary segmental glomerulosclerosis and associated acute tubular injury was made. There were no symptoms or laboratory abnormalities to support extrarenal manifestations or systemic TMA; thus, renal-limited TMA was the primary diagnosis. A review of the patient’s medication list, including over-the-counter supplements, did not show any quinines or other drugs known to cause TMA.

PLD chemotherapy was discontinued, and although the AKI worsened initially (sCr 4.35 mg/dL), requiring acute hemodialysis, some renal recovery was noted 2 months later. The patient was taken off hemodialysis for about 2 months. However, at the time of this report, palliative hemodialysis was reinitiated twice weekly for volume management, given a 24-hour creatinine clearance of ∼5 to 8 mL/min, and the patient was noted to have symptomatic volume overload. The patient also elected to discontinue further PLD treatment in lieu of conservative management for Kaposi sarcoma, a comfort care approach.

## Discussion

This case highlights an 80-year-old man who developed AKI and significant proteinuria because of biopsy-confirmed kidney-limited TMA after a relatively low cumulative exposure to PLD. The temporal relationship between PLD therapy and the onset of kidney dysfunction, without an alternative identified etiology, strongly suggests PLD as the primary causative agent. Other rarer triggers, such as complement-mediated TMA, atypical hemolytic uremic syndrome, or cobalamin C deficiency, were negative on additional genetic testing. Castleman disease, another human herpesvirus 8-associated condition linked to kidney-limited microangiopathy, was considered but deemed unlikely given the absence of splenomegaly, hepatomegaly, or generalized lymphadenopathy. Finally, although the patient had diabetes and hypertension—both associated with secondary segmental glomerulosclerosis and mesangial sclerosis seen on biopsy—the prominent features of chronic active endothelial injury, such as glomerular capillary wall double contours, are not typical of these conditions.

TMA is a pathological process involving endothelial cell damage in arterioles and capillaries, frequently associated with thrombosis.^5^ It is typically inferred from clinical signs, including microangiopathic hemolytic anemia, thrombocytopenia, and AKI. A biopsy is required for diagnosis of isolated renal-limited TMA.

Encapsulation of doxorubicin in liposomes enhances its therapeutic efficacy by improving tumor targeting and reducing the severity of adverse effects, including cardiotoxicity.^6^ Although nonliposomal anthracyclines have been linked to kidney conditions such as minimal change disease, focal segmental glomerulosclerosis, and collapsing glomerulopathy, neither conventional doxorubicin nor PLD has been strongly associated with TMA findings on kidney biopsy.^7^^,^^8^ It has previously been reported that 23% of 56 patients treated by PLD (median cumulative dose 470 mg/m^2^, median follow-up time of 50 months), either by itself or with other anticancer drugs, developed stage 3-4 chronic kidney disease and hypertension; however, kidney biopsies were not performed.^9^

Reports of PLD-associated TMA are rare, often confounded by concurrent exposure to other TMA-inducing agents. Shavit et al^9^ described 3 patients with biopsy-confirmed kidney-limited TMA after high cumulative doses of PLD (880-1,445 mg/m^2^); however, 2 of the 3 had also received bevacizumab or gemcitabine, also known to cause TMA. All 3 patients experienced AKI stage 3, with an increase in sCr from a normal baseline of 0.9 (±0.2) mg/dL to 5.2 (±0.3) mg/dL. All 3 patients also presented with worsening hypertension and subnephrotic range proteinuria, which improved partially on PLD withdrawal.^9^ Rodriguez-Ramirez et al^10^ described a kidney transplant recipient with Kaposi sarcoma who developed kidney-limited TMA while on PLD, with an increase in sCr from 2.2 to 3.4 mg/dL. The patient had also been exposed to gemcitabine and sirolimus, both known to cause TMA. After discontinuing PLD, sCr returned to baseline, although sirolimus was continued. Because of a prior good response to PLD, treatment was reinitiated. Three months later (cumulative dose 800 mg/m^2^), the patient’s kidney function worsened (sCr 4.2 mg/dL), along with hypertension and subnephrotic proteinuria, prompting PLD withdrawal. Glezerman et al^11^ reported 2 cases of biopsy-proven TMA in patients on PLD monotherapy, both with solitary kidneys and presenting with elevated sCr, proteinuria, and edema. One patient (cumulative dose 760 mg/m^2^) had a sustained sCr increase from 1.0 to 1.8 mg/dL despite PLD withdrawal. The other (cumulative dose 1,240 mg/m^2^) showed a more severe increase in sCr from 1.1 to 5.4 mg/dL, with partial recovery to 3.4 mg/dL after stopping PLD.

The exact mechanism of PLD-induced TMA remains unclear, although reactive oxygen species-mediated endothelial injury and platelet aggregation are likely contributors.^12^ This case is notable because TMA developed at a cumulative dose of ∼300 mg/m^2^—lower than previously reported thresholds of 760-1,445 mg/m^2^.^11^ This report emphasizes the need for close monitoring of patients on PLD therapy, particularly those with hypertension, hematuria, proteinuria, or unexplained renal dysfunction. Routine evaluations of kidney function, hemoglobin, platelet counts, and lactate dehydrogenase levels are essential, with early biopsy consideration when reversible causes are absent. Documenting this case adds to the growing evidence needed to refine guidelines and improve understanding of PLD therapy and TMA diagnosis and treatment.
